# Evaluation of an air quality warning system for vulnerable and susceptible individuals in Korea: an interrupted time series analysis

**DOI:** 10.4178/epih.e2023020

**Published:** 2023-02-14

**Authors:** YouHyun Park, Jun Hyuk Koo, Hoyeon Jeong, Ji Ye Jung, Changsoo Kim, Dae Ryong Kang

**Affiliations:** 1 Department of Biostatistics, Graduate School of Yonsei University, Seoul, Korea; 2 National Health Big Data Clinical Research Institute, Yonsei University Wonju Industry-Academic Cooperation Foundation, Wonju, Korea; 3 Division of Pulmonary and Critical Care Medicine, Department of Internal Medicine, Severance Hospital, Yonsei University College of Medicine, Seoul, Korea; 4 Department of Preventive Medicine, Yonsei University College of Medicine, Seoul, Korea; 5 Department of Precision Medicine, Yonsei University Wonju College of Medicine, Wonju, Korea

**Keywords:** Air quality, Alert system, Environmental policy

## Abstract

**OBJECTIVES:**

This study was conducted to elucidate the effects of an air quality warning system (AQWS) implemented in January 2015 in Korea by analyzing changes in the incidence and exacerbation rates of environmental diseases.

**METHODS:**

Data from patients with environmental diseases were extracted from the National Health Insurance Service-National Sample Cohort database from 2010 to 2019, and data on environmental risk factors were acquired from the AirKorea database. Patient and meteorological data were linked based on residential area. An interrupted time series analysis with Poisson segmented regression was used to compare the rates before and after AQWS introduction. Adjustment variables included seasonality, air pollutants (carbon monoxide, nitrogen dioxide, sulfur dioxide, particulate matter less than 10 μm in diameter, and ozone), temperature, and humidity.

**RESULTS:**

After AQWS implementation, the incidence of asthma gradually decreased by 20.5%. Cardiovascular disease and stroke incidence also significantly decreased (by 34.3 and 43.0%, respectively). However, no immediate or gradual decrease was identified in the exacerbation rate of any environmental disease after AQWS implementation. Sensitivity analyses were performed according to age, disability, and health insurance coverage type. Overall, the AQWS effectively mitigated the occurrence of most environmental diseases in Korea. However, the relationships between alarm system implementation and reduced incidence differed among diseases based on the characteristics of vulnerable and sensitive individuals.

**CONCLUSIONS:**

Our results suggest that by tailoring the AQWS to demographic and sociological characteristics and providing enhanced education about the warning system, interventions can become an efficient policy tool to decrease air pollution-related health risks.

## INTRODUCTION

The physical environment has been suggested to be one of the most important determinants of health [1]. Indoor and outdoor air pollution are adverse conditions of the physical environment and harm human health upon exposure. A 10 μg/m^3^ increase in fine dust with a diameter less than 10 µm (PM_10_), a representative air pollutant, has been shown to exacerbate the all-cause daily mortality by 0.2% to 0.6% globally [2], with similar results in Korea [1,3]. Moreover, numerous studies have demonstrated that air pollution is a risk factor for the development of various diseases, including cardiovascular, respiratory, endocrine, and musculoskeletal diseases [4-8]. Currently, indoor and outdoor air pollution cause over 6 million annual deaths worldwide [9]. Moreover, approximately 99% of humans breathe air containing levels of pollutants that exceed the World Health Organization (WHO) air quality standards. Thus, an urgent need exists to mitigate and control air pollution at the global level.

In this context, at the 69th World Health Assembly, the WHO presented a draft global response roadmap with 4 components: (1) expansion of the knowledge base, (2) monitoring and reporting, (3) global leadership and coordination, and (4) strengthening of the institutional capacity to respond to the adverse health effects of air pollution [10]. The roadmap was introduced to secure evidence of the adverse health effects of air pollution by establishing relevant national and subnational urban policies such as health impact assessment, monitoring, public awareness improvement, and health action planning. As such, the management of air pollution at the global level can be facilitated. Since 1987, the WHO has regularly outlined international guidelines and standards for air quality [11]. At the same time, national governments have monitored air pollution by analyzing air quality index (AQI) values according to country-specific characteristics [12], enabling the use of AQI metrics to operate air quality forecasting and warning systems. Many countries, including the United States, the United Kingdom, Canada, China, and Korea, have successfully introduced such systems based on AQIs.

Although the corpus of studies focused on these forecasting and warning systems is growing, evidence regarding their effectiveness is still limited. A study in Santiago, Chile indicated that an intervention program conducted on days with severe air pollution effectively reduced air pollution and mortality in the short term [1]. A separate study in Korea demonstrated that a mobile, text messaging-based warning system also facilitated the reduction of respiratory diseases [12]. However, a study conducted in Paris, France showed no statistically significant relationship between the use of an alert system and mortality from respiratory diseases [14]. Two other studies, conducted in Hong Kong, reported that an air quality health index program yielded a significant reduction in hospitalizations for certain diseases among children and the elderly. However, those authors reported no statistically significant differences in the number of hospitalizations for respiratory and cardiovascular diseases in the general population [15,16]. A study conducted in Toronto, Canada revealed some significant effects but concluded that the effectiveness of the alert program alone was limited [17]. In this context, Korea introduced the Air Quality Conservation Act in January 2015, potentially impacting the numbers of inpatients and outpatients due to 4 representative environmental diseases (chronic obstructive pulmonary disease [COPD], asthma, cardiovascular disease, and stroke). However, no studies have addressed the statistical relationship between the air quality warning system (AQWS) in Korea and those numbers, even among groups particularly vulnerable to air pollution. Groups sensitive or vulnerable to pollution traditionally include the elderly, newborns and infants, pregnant women, people with allergies, people with lung and/or heart disease, and the poor [18,19].

This study elucidated the effect of the air quality alert system of Korea by analyzing changes in the incidence rates of environmental diseases among target groups including children, the elderly, and residents of industrial complexes. To this end, we utilized an interrupted time series (ITS) analysis with Poisson segmented regression to identify changes in the incidence rates of environmental diseases after implementation of an AQWS in Korea using data from 2010 to 2019.

## MATERIALS AND METHODS

### Data sources

Korea has maintained a national health insurance system since 1963 under the Korean National Health Insurance Service (NHIS). Nearly all related data from the health system are centralized in large databases. The sample size of the NHIS-National Sample Cohort (NSC) database is approximately 1 million, comprising 2% of randomly selected Koreans who had met the qualifications for at least 1 year as of December 2006. To secure representation of the Korean population, stratified sampling was performed considering sex, age, income level, and region. Registered participants were monitored from January 1, 2002 to December 31, 2019. The NHIS-NSC data include a unique, anonymous number for each patient and summarizes age; sex; type of insurance; a list of diagnoses according to the International Classification of Diseases, 10th revision; medical costs claimed; and prescribed medications. A detailed explanation of the NHIS-NSC can be found in Lee et al. [20].

AirKorea (https://www.airkorea.or.kr/eng) provides daily concentrations of certain air pollutants per minute (PM_10_, nitrogen dioxide [NO_2_], sulfur dioxide [SO_2_], carbon monoxide [CO], and ozone [O_3_]), measured at the administrative unit (*si* [city], *gun* [county], and *gu* [district]) level. The measurements have been performed at air quality monitoring stations operated by the Korean Ministry of Environment since 2001. In 2015, particulate matter with a diameter less than 2.5 µm (PM_2.5_) concentration began to be measured, but it was excluded from this analysis because the study period began in 2010. PM_10_ and PM_2.5_ concentrations were measured using β-ray absorption, NO_2_ concentrations using chemiluminescence, SO_2_ using ultraviolet fluorescence, CO using the non-dispersive infrared method, and O_3_ using the ultraviolet photometric method, according to the Standards of Measuring Air Pollutants of Korea. AirKorea adhered to quality assurance procedures for the measurements and data collection [21].

### Study population

In total, 1 million Koreans were sampled in the NHIS-NSC. Among these records, for each year between 2010 and 2019, we selected the data from individuals who had outpatient or hospitalization claims due to environmental diseases. To evaluate incidence, we selected patients with a primary or secondary diagnosis of environmental disease [22] including COPD (ICD10 J42, J44 and J431-J439) [8], asthma (ICD10 J45-46) [23,24], stroke (ICD10 I60-I63) [6], and cardiovascular disease (ICD10 I47: paroxysmal tachycardia, I48: atrial fibrillation, I49: arrhythmias, and I50: heart failure) [4,16,25,26] each year from 2010 to 2019. We defined exacerbation based on the hospital admissions or emergency room visits due to environmental diseases in each year during that period. Additionally, we considered chronic digestive disease (ICD10 K21, K522, K29-30, K72-76, K80, K811, and K900) as a comparison group because people with digestive disease would be unlikely to respond to air quality health index warnings [15,26], as such warnings mainly concern people with respiratory or cardiovascular diseases. Since susceptibility may vary depending on demographic and sociological characteristics, a sensitivity analysis was performed considering age (children, < 15; adults, 15-60; and the elderly, ≥ 60 years), disability (yes/no), and applicable types of health care systems (medical aid beneficiaries/health insurance beneficiaries).

### Outcomes

The outcomes considered were the monthly age-standardized incidence rate and the exacerbation rate (indicated by hospital admissions or emergency room visits) of environmental diseases by administrative unit from 2010 to 2019. The crude incidence and exacerbation rates were calculated using the number of monthly patients per million of the population with environmental diseases, with the administrative unit as the numerator and the total population (per the National Statistical Office) for the relevant year as the denominator. Both the incidence and exacerbation rates of environmental diseases were indirectly age-standardized using the Korean resident registration population of 2005 as the standard population.

### Air pollutants and meteorological data

We calculated the monthly mean concentrations of air pollutants (PM_10_, NO_2_, SO_2_, CO, and O_3_) and meteorological data (temperature and humidity) for each administrative unit by linking the NHIS-NSC and AirKorea data based on residential area. Furthermore, we utilized the NHIS-NSC to identify the residential addresses of all participants for each year at the administrative unit level. The daily average concentrations of air pollutants and meteorological data in all regions were calculated using the AirKorea data. The concentration of each air pollutant and meteorological data were associated with the residential address at the corresponding time, and the monthly mean concentrations for the entire follow-up period were calculated.

### Statistical analysis

In this study, we used ITS analysis with Poisson segmented regression for the period from 2010 to 2019 to identify changes in the incidence and exacerbation rates of environmental diseases before and after the introduction of AQWS (January 2015). The Poisson segmented regression was adjusted for seasonality, and the targeted air pollutants (CO, NO_2_, SO_2_, PM_10_, and O_3_), temperature, and humidity were considered. The findings were validated using digestive diseases as a control group. The ITS model used in this study [25] is detailed in Supplementary Material 1.

**Figure 1.**
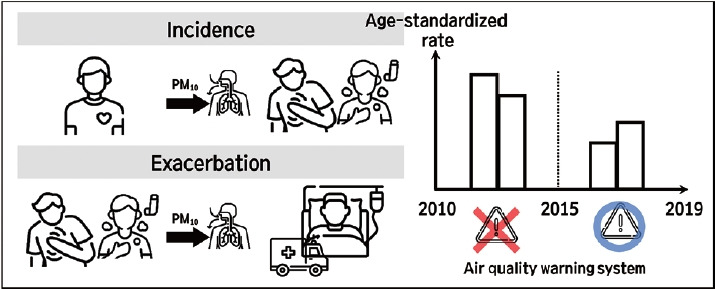
Study design. PM_10_, particulate matter with a diameter less than 10 μm.

We estimated the relative risks (RRs) and 95% confidence intervals (CIs) for the associations between AQWS implementation and the development of environmental diseases. All statistical analyses were performed using SAS version 9.4 (SAS Institute Inc., Cary, NC, USA). A p-value < 0.05 was considered to indicate statistical significance. The study design is illustrated in Figure 1.

### Ethics statement

This study was approved by the Institutional Review Board of Wonju Severance Christian Hospital (CR321327). Because it was a retrospective study conducted using anonymous claims data, the requirements for informed consent were waived.

## RESULTS

### Participants’ characteristics

Differences between the annual incidence and exacerbation rates of environmental diseases before and after 2015, when the fine dust warning system was implemented, were assessed using the t-test (Table 1). The age-standardized incidence rates of environmental diseases were lower after the introduction of the AQWS than before, except for cardiovascular diseases, and all results were statistically significant (incidence rate per 1,000,000 population from before to after implementation: COPD, 91.21 to 76.64; asthma, 516.56 to 365.92; cardiovascular disease, 72.95 to 78.32; stroke, 121.63 to 110.68; digestive disease, 2802.58 to 2787.80). Furthermore, the age-standardized exacerbation rates of environmental diseases were lower after the introduction of AQWS than before, except for cardiovascular and digestive diseases; most of those results were statistically significant (exacerbation rate per 10,000,000 population from before to after implementation: COPD, 42.88 to 41.61; asthma, 97.86 to 80.94; cardiovascular disease, 46.36 to 50.61; stroke, 186.64 to 18.67; digestive disease, 626.84 to 737.97).

The median annual concentrations of CO, NO_2_, SO_2_, PM_10_, and O_3_ were 532.87 μg/m^3^ (interquartile range [IQR], 447.44 to 647.42), 36.34 μg/m^3^ (IQR, 27.55 to 46.46), 11.40 μg/m^3^ (IQR, 8.54 to 14.63), 44.00 μg/m^3^ (IQR, 34.94 to 53.00) and 53.08 μg/m^3^ (IQR, 39.37 to 67.70), respectively. All of the gaseous and particulate air pollutants except O_3_ increased after 2015 relative to before 2015, and the differences were statistically significant. The monthly median ambient temperature and relative humidity over the entire study period were 14.00°C and 72.00%, respectively. No statistically significant difference was discerned between pre-implementation and post-implementation of the fine dust warning system.

### Interrupted time series analysis for air quality warning system

**Figure 2.**
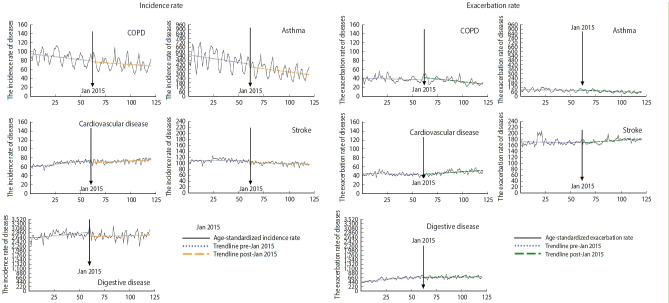
Time series plot of monthly mean, and age-standardized emergency hospital admissions for environmental diseases in Korea between 2010 and 2019.

Figure 2 displays the immediate and gradual effects of AQWS implementation on the rates of environmental diseases. After implementation of the AQWS, the incidence of asthma gradually decreased by 20.5% (RR, 0.795; 95% CI, 0.725 to 0.872) (Table 2). Additionally, the incidence rates of cardiovascular disease and stroke significantly decreased by 34.3% and 43.0%, respectively (cardiovascular disease: RR, 0.657; 95% CI, 0.471 to 0.916; stroke: RR, 0.570; 95% CI, 0.344 to 0.944).

However, no immediate or gradual decrease was identified in the exacerbation rate for any environmental disease after implementation of the AQWS, despite the metrics for COPD (RR, 0.971; 95% CI, 0.937 to 1.006), asthma (RR, 0.937; 95% CI, 0.846 to 1.038), cardiovascular disease (RR, 1.014; 95% CI, 0.956 to 1.043), and stroke (RR, 1.000; 95% CI, 0.938 to 1.067). Furthermore, the gradual post-policy changes were somewhat unremarkable for all analyzed diseases.

### Sensitivity analyses

We also performed sensitivity analyses according to age, with the age-stratified estimates summarized in Supplementary Materials 2 and 3. After AQWS implementation, the only significant immediate reduction in the incidence rate was observed in the age group excluding children (age < 15 years). In children, the incidence rate of asthma alone exhibited a significant gradual decrease. The diseases immediately impacted by AQWS implementation differed by age group. For instance, we identified an immediate reduction in the incidence rate among young and middle-aged adults for both asthma (RR, 0.598; 95% CI, 0.390 to 0.917) and cardiovascular disease (RR, 0.046; 95% CI, 0.013 to 0.170), along with a decrease in the incidence rate of stroke in the elderly (age ≥ 60 years; RR, 0.004; 95% CI, 0.001 to 0.268). Moreover, we observed a gradual impact on the incidence rate of asthma in the age group including children, with decreases of 12.7%, 3.5%, and 42.1%. The greatest decrease was identified in the elderly, whereas the smallest decrease was identified in young and middle-aged adults. The exacerbation rate of environmental disease in susceptible and vulnerable groups was not statistically significant for either immediate or gradual changes.

We also performed sensitivity analyses for persons with disabilities, as they may be more adversely impacted by environmental risk factors than non-disabled people [23] (Supplementary Material 4). In the results, only COPD and asthma, the most well-known environmental respiratory diseases, exhibited statistically significant gradual decreases in incidence after the implementation of AQWS relative to the prior period. Although the patterns were similar for non-disabled and disabled people, an immediate effect on asthma and stroke was also discerned for the latter group (Supplementary Material 5).

Moreover, the analysis by type of health insurance coverage demonstrated that recipients in the medical aid program experienced a gradual decrease in the incidence of asthma and an immediate decrease in the incidence of cardiovascular disease. The analysis of those insured under the National Health Insurance system also revealed an associated gradual decrease in respiratory disease occurrence. Overall, these results confirm an association with an immediate decrease in asthma, cardiovascular disease, and stroke. The policy impact by type of health insurance coverage is detailed in Supplementary Materials 6 and 7.

## DISCUSSION

In this study, the implementation of an AQWS in Korea was associated with immediate reductions in cardiovascular disease and stroke and a gradual reduction in asthma. Unlike other age groups, for the elderly, the implementation of the AQWS was attributed to a gradual reduction in COPD. Moreover, it was frequently associated with an immediate reduction in the incidence of environmental diseases, particularly in groups that were not particularly vulnerable to exposure to environmental hazards, as opposed to those that were potentially more vulnerable, such as the elderly and those with disabilities. At the same time, we found that the implementation of the fine dust warning system had no distinct effect on the exacerbation of environmental diseases, namely on visits or hospitalizations of existing environmental disease patients in the emergency room. These findings align with those of previous studies, which indicate that the presence of an air pollution alert system is not associated with the deterioration of existing respiratory or heart disease patients [15-17,23].

Countries such as the United Kingdom, Canada [17], the United States, and Hong Kong [15] have begun to implement air pollution warning systems. Likewise, in Korea, an AQWS was introduced to encourage changes in the behavior patterns of the at-risk population by warning the public about the risks of air pollution [28]. According to Radisic et al. [29], the health effects of these policies are realized when at-risk groups decrease their health risks by reducing exposure to air pollution via changes in their behavior in response to such information. Neidell & Kinney [23] studied the effect of air quality alarms on outdoor activities; although they did not confirm the influence of such alarms on health, their influence on human behavior was reported.

Previous studies have also indicated that demographic and sociological characteristics, perception of individual risk, and knowledge of air pollution are important factors related to AQWS. For instance, Radisic et al. [29] suggested that people who lack awareness of the health impact of air pollution and those who are less educated and informed about these issues (typically the elderly) often fail to make behavioral changes because they do not comply with the information. The present findings are consistent with those of previous studies. In particular, we revealed a clear policy effect, but it depended in part on the characteristics of the vulnerable population. The policy effect was insignificant in children [24], the elderly [30-32], disabled individuals [25], and Medicaid recipients [24,33], who constitute populations that would be arguably particularly vulnerable to air pollution.

The elderly may not immediately respond to new information [29], and it is well known that children and some elderly populations have relatively low risk cognitive levels [34]. According to Durand et al. [35], Medicaid recipients are characterized by low health literacy. Additionally, one study [36] previously reported that individuals outside of at-risk groups exhibited higher perceptions toward risk of air pollution than those included in at-risk groups. In Korea, a mobile-based wireless emergency alert system was utilized as an air pollution alert system [37].

This system sends text messages to all mobile devices within the coverage area of the monitoring station for harmful environmental factors [38]. These text-based alarm services can immediately inform people of potential threats faced at their current location. However, the level of risk was delivered in the form of messages. Such a general warning of risk does not cause direct behavior changes in vulnerable populations. According to Kalyanaraman & Sundar [39], personalized warnings are more effective in altering individual health behavior. Moreover, while interventions involving personalized risk information impact some health-related behaviors, they do not affect the understanding or perception of risk [40]. Thus, it is reasonable to suggest that by only issuing air quality alerts, one can achieve only limited public health effects. This potentially justifies the implementation of compulsory public measures to reduce air pollution on severe days [17]. If education about the policies and diversity in alert methods are simultaneously introduced, an AQWS could have a greater impact on the health improvement of the people.

In this study, for the first time, we reported the impact of an AQWS on the incidence or exacerbation of environmental diseases in Korea by elucidating previously unexplored aspects of this topic. First, this study was not limited to the occurrence of respiratory diseases, which are arguably deemed to be most strongly related to pollution, but also included environmental diseases such as cardiovascular disease and stroke. Moreover, it involved a stratified analysis of the impact of AQWS for the first time. Our results were uniquely derived using quasi-experimental methods and ITS analysis [41]. These methods were chosen because randomized controlled research was not possible as a research model to best evaluate these policy effects [42]. In particular, ITS was most suitable for the design of this study, because it allowed for stratified analysis. This analysis was used to evaluate the differential impact of intervention or policy change on individual subgroups [43,44]. Although ITS can be slightly hindered by the inherent lack of a control group, we attempted to strengthen the validity of the findings using the negative control results to detect unknown time-varying confounders [45].

Despite the advances reported in this study, limitations remain. First, as we used monthly aggregated data (on the rates of incidence and exacerbation) rather than individual data, we could not estimate individual levels of causal inference. However, as the purpose of this study was to evaluate a policy, the causal inference was made at the popular rather than the individual level. Second, although the influence of the AQWS on environmental diseases was clearly revealed, compliance with the AQWS was not reflected in our study; in other words, it is still unclear whether the public accessed and utilized the relevant information. Moreover, the awareness level regarding the air quality health index was not considered. In the future, qualitative studies should be accompanied by recognition of and compliance with fine dust warning systems, and the vectors for potential policy improvement should be identified.

Overall, the study results show that the AQWS was effective in mitigating the occurrence of most environmental diseases in Korea. However, the relationships between alarm system implementation and reduced incidence differed among diseases based on the characteristics of vulnerable and sensitive individuals. Our results suggest that through tailored diversification of the AQWS by demographic and sociological characteristics as well as enhanced education about the warning system, such intervention can be an efficient policy tool to prevent health risks from air pollution.

## Figures and Tables

**Table 1.** Summary of age-standardized monthly mean^1^ hospital admissions (daily count) for environmental diseases^2^, covariates, and meteorological variables before and after AQWS implementation and for the entire study period (2010-2019)

Variables		Total 2010-2019	Before 2010-2014	After 2015-2019	p-value
Incidence rate					
	COPD	82.87 (64.9, 109.04)	91.21 (70.85, 119.03)	76.64 (60.19, 100.18)	<0.001
	Asthma	433.92 (335.01, 543.34)	516.56 (417.19, 621.35)	362.92 (289.56, 447.73)	<0.001
	Cardiovascular disease	75.16 (60.02, 97.39)	72.95 (58.49, 94.98)	78.32 (61.56, 99.73)	<0.001
	Stroke	115.65 (88.2, 139.31)	121.63 (93.43, 142.81)	110.68 (82.87, 133.46)	<0.001
	Digestive disease^3^	2,794.03 (2,530.3, 3,100.62)	2,802.58 (2,530.3, 3,145.03)	2,787.8 (2,530.06, 3,070.66)	0.017
Exacerbation rate					
	COPD	42.45 (24.86, 63.81)	42.88 (25.22, 66.79)	41.61 (24.48, 62.3)	0.013
	Asthma	89.63 (58.25, 133.34)	97.86 (67.3, 145.81)	80.94 (49.66, 119.25)	<0.001
	Cardiovascular disease	48.41 (34.97, 67.07)	46.36 (33.69, 62.51)	50.61 (36.06, 71.15)	<0.001
	Stroke	185.36 (147.56, 224.16)	186.64 (147.13, 222.37)	183.67 (148.24, 226.94)	0.255
	Digestive disease^3^	680.48 (561.43, 825.40)	626.84 (511.25, 750.71)	737.97 (631.86, 876.53)	<0.001
Covariates					
	CO	532.87 (447.44, 647.42)	543.23 (461.66, 662.35)	524.02 (431.6, 638.99)	<0.001
	NO_2_	36.34 (27.55, 46.46)	38.51 (29.27, 48.49)	34.47 (26.00, 44.85)	<0.001
	SO_2_	11.40 (8.54, 14.63)	12.76 (9.37, 16.48)	10.52 (7.92, 12.98)	<0.001
	PM_10_	44.00 (34.94, 53.00)	45.69 (36.50, 55.51)	42.22 (33.45, 50.95)	<0.001
	O_3_	53.08 (39.370, 67.70)	49.43 (37.60, 64.98)	56.13 (41.19, 71.37)	<0.001
Meteorological variables					
	Temperature	14.00 (5.30, 21.80)	13.60 (4.60, 21.90)	14.40 (5.75, 21.60)	0.143
	Relative humidity	72.00 (63.00, 80.00)	72.00 (64.00, 79.50)	73.00 (63.00, 81.00)	0.210

Values are presented as median (interquartile range). AQWS, air quality warning system; COPD, chronic obstructive pulmonary disease; CO, carbon monoxide; NO2, nitrogen dioxide; SO2, sulfur dioxide; PM10, particulate matter with a diameter less than 10 μm; O3, ozone.

1 Monthly mean concentration of respiratory diseases in Hong Kong, age-standardized based on the World Health Organization standard population per 1,000,000. Hong Kong’s population at the end of 2013 was 7.2 million.

2 Environmental diseases: COPD, asthma, heart failure, stroke.

3 Digestive disease: control disease for the study, excluding peptic ulcer diseases.

**Table 2.** Immediate and gradual changes in the incidence of environmental diseases after implementation of an air quality warning system based on multivariate analysis^1^

Environmental disease^2^	Incidence rate		Exacerbation rate	
	Immediate effects	Gradual effects	Immediate effects	Gradual effects
COPD	0.78 (0.42, 1.44)	0.98 (0.95, 1.00)	1.76 (0.79, 3.93)	0.97 (0.94, 1.01)
Asthma	0.23 (0.05, 1.22)	0.80 (0.73, 0.87)^***^	0.97 (0.21, 4.47)	0.94 (0.85, 1.04)
Cardiovascular disease	0.66 (0.47, 0.92)^***^	1.01 (0.99, 1.03)	1.10 (0.75, 1.61)	1.01 (0.99, 1.04)
Stroke	0.57 (0.34, 0.94)^***^	0.98 (0.95, 1.02)	1.79 (0.40, 8.06)	1.00 (0.94, 1.07)
Digestive disease^3^	0.50 (0.00, 1.00)	0.77 (0.57, 1.05)	0.50 (0.00, 1.00)	1.07 (0.85, 1.34)

Values are presented as relative risk (95% confidence interval). COPD, chronic obstructive pulmonary disease.

1 Age-standardized based on the Korean standard population in 2005; Adjusted for seasonality, temperature, humidity, time trend, and concentrations of carbon monoxide, sulfur dioxide, nitrogen dioxide, particulate matter with a diameter less than 10 μm, and ozone.

2 Environmental diseases: COPD, asthma, heart failure, stroke.

3 Digestive disease: control disease for the study, excluding peptic ulcer diseases.

*** p<0.001.

## SUPPLEMENTARY MATERIALS

Supplementary Material 1.

Supplementary Material 2. Immediate and gradual changes for incidence of environmental diseasesa after implementation of the Air Quality Warning System in multivariate analysisb by age.

Supplementary Material 3. . Time series plot of monthly mean, age standardized emergency hospital admissions for environmental diseases by age in Korea during 2010–2019.

Supplementary Material 4. Immediate and gradual changes for incidence of environmental diseasesa after implementation of the Air Quality Warning System in multivariate analysisb by disability.

Supplementary Material 5. Time series plot of monthly mean, age standardized emergency hospital admissions for environmental diseases by disability in Korea during 2010–2019.

Supplementary Material 6. Immediate and gradual changes for incidence of environmental diseasesa after implementation of the Air Quality Warning System in multivariate analysisb by types of health care systems applicable.

Supplementary Material 7. Time series plot of monthly mean, age standardized emergency hospital admissions for environmental diseases by types of health care systems applicable in Korea during 2010–2019.
