# Supplementary material for: Evaluation of an air quality warning system for vulnerable and susceptible individuals in Korea: an interrupted time series analysis
Source: Epidemiol Health. 2023 Feb 14;45:e2023020. doi: 10.4178/epih.e2023020 (PMC10581892; doi:10.4178/epih.e2023020)
Supplement: Supplementary Material 1. [file epih-45-e2023020-Supplementary-1.docx]

**Supplementary Materials**

**An evaluation of the air quality warning system for vulnerable and susceptible individuals in Korea: an interrupted time series analysis**

**YouHyun Park^1,2^, Koo Jun Hyuk^2^, Hoyeon Jeong^1,2^, Ji Ye Jung^3^, Changsoo Kim^4^, Dae Ryong Kang^2,5^**

*^1^**Department of Biostatistics, Graduate School of Yonsei University, Seoul, Korea;*

*^2^National Health Big Data Clinical Research Institute, Yonsei University Wonju Industry-Academic Cooperation Foundation, Wonju, Korea*

*^3^Division of Pulmonary and Critical Care Medicine, Department of Internal Medicine, Severance Hospital, Yonsei University College of Medicine, Seoul, Korea;*

*^4^Department of Preventive Medicine, Yonsei University College of Medicine, Seoul, Korea;*

*^5^Department of Precision Medicine, Wonju College of Medicine, Yonsei University, Wonju, Korea*

**Supplementary Material 1.**

ITS analysis is an analytical methodology based on the experimental approach, which can be effectively used for identifying the temporal effects of interventions in time series data or cohorts before and after intervention. In particular, it is a model that estimates different intercepts and slopes in the pre-intervention and post-intervention time interval. Thus, one can use this model to estimate the amount of temporal change in the medical use over time while also correcting the persistent trends and potential autocorrelation of time series data. The ITS model used in this study [22] is represented by Eq. (1):

$Y_{t}=\beta_{0}+\beta_{1}*\mathrm{Time}_{t}+\beta_{2}*{Intervention}_{t}+\beta_{3}*{TimeafterPolicy}_{t}+\varepsilon_{t}$.

(1)

where β0 is the incidence/exacerbation rate of environmental diseases in the January 2010, when the analysis started; β1 is the amount of change in monthly mean, age-standardized incidence/exacerbation rate according to the lapse of unit time before the introduction of AQWS; β2 is the immediate changes after the introduction of AQWS (immediate intervention effect); β3 is the trend in changes in reflection of the intervention effect since the introduction of AQWS, compared with the monthly trend before intervention (trend); (β1+ β3) is gradual changes after intervention; Timet is the number of consecutive monthly units in the period in which the corresponding time series data belong; Interventiont is the time of introduction of AQWS in Korea (January 2015); TimeafterPolicyt is the time after the introduction of AQWS; εt is the error term; and t is the number of months from the 1st month of 2015 to the last month of 2019.
