# Supplementary material for: Evaluation of an air quality warning system for vulnerable and susceptible individuals in Korea: an interrupted time series analysis
Source: Epidemiol Health. 2023 Feb 14;45:e2023020. doi: 10.4178/epih.e2023020 (PMC10581892; doi:10.4178/epih.e2023020)
Supplement: Supplementary Material 2. — Immediate and gradual changes for incidence of environmental diseasesa after implementation of the Air Quality Warning System in multivariate analysisb by age. [file epih-45-e2023020-Supplementary-2.docx]

**Supplementary Materials**

**An evaluation of the air quality warning system for vulnerable and susceptible individuals in Korea: an interrupted time series analysis**

**YouHyun Park^1,2^, Koo Jun Hyuk^2^, Hoyeon Jeong^1,2^, Ji Ye Jung^3^, Changsoo Kim^4^, Dae Ryong Kang^2,5^**

*^1^**Department of Biostatistics, Graduate School of Yonsei University, Seoul, Korea;*

*^2^National Health Big Data Clinical Research Institute, Yonsei University Wonju Industry-Academic Cooperation Foundation, Wonju, Korea*

*^3^Division of Pulmonary and Critical Care Medicine, Department of Internal Medicine, Severance Hospital, Yonsei University College of Medicine, Seoul, Korea;*

*^4^Department of Preventive Medicine, Yonsei University College of Medicine, Seoul, Korea;*

*^5^Department of Precision Medicine, Wonju College of Medicine, Yonsei University, Wonju, Korea*

Supplementary Material 2. Immediate and gradual changes for incidence of environmental diseases^a^ after implementation of the Air Quality Warning System in multivariate analysis^b^ by age.

| Environmental  diseases^*^ | **<15 Years** | | **15-60 Years** | | **>60 Years** | |
| --- | --- | --- | --- | --- | --- | --- |
|  | Immediate Effects | Gradual Effects | Immediate Effects | Gradual Effects | Immediate Effects | Gradual Effects |
|  | RR^*^ (95% CI) | RR (95% CI) | RR (95% CI) | RR (95% CI) | RR (95% CI) | RR (95% CI) |
| **Incidence Rate** | |  |  |  |  |  |
| COPD | 1.66 (0.31-8.99) | 1.04 (0.92-1.16) | 0.51 (0.19-1.88) | 0.97 (0.85-1.12) | 0.11 (0.00-2.63) | **0.80 (0.67-0.95)**^‡^ |
| Asthma | 0.43 (0.08-2.19) | **0.87 (0.81-0.95)**^‡^ | **0.60 (0.39-0.92)**^‡^ | **0.97 (0.94-0.99)**^‡^ | 0.44 (0.01-3.75) | **0.58 (0.45-0.75)**^‡^ |
| Cardiovascular disease | 0.84 (0.64-1.10) | 1.01 (0.99-1.02) | **0.05 (0.01-0.17)**^‡^ | 1.07 (0.95-1.20) | 0.14 (0.02-1.23) | 1.04 (0.86-1.24) |
| Stroke | 2.76 (0.17-4.52) | 1.01 (0.93-1.09) | 0.45 (0.05-3.69) | 0.99 (0.88-1.12) | **0.04 (0.01-0.24)**^‡^ | 0.87 (0.65-1.15) |
| Digestive disease^*^ | - | 0.56 (0.33-0.95) | - | 1.46 (0.20-10.81) | - | 0.20 (0.04-1.02) |
| **Exacerbation Rate** | |  |  |  |  |  |
| COPD | 1.30 (0.96-1.76) | 0.99 (0.98-1.01) | 1.19 (0.97-1.45) | 0.99 (0.99-1.00) | 1.20 (0.63-2.29) | 0.98 (0.95-1.04) |
| Asthma | 1.16 (0.49-2.70) | 0.96 (0.92-1.01) | 1.12 (0.73-1.73) | 0.99 (0.97-1.01) | 1.23 (0.81-1.88) | 0.98 (0.95-1.01) |
| Cardiovascular disease | 0.97 (0.83-1.13) | 1.00 (0.99-1.01) | 0.86 (0.74-1.01) | 1.00 (0.99-1.01) | 1.22 (0.86-1.74) | 1.01 (0.99-1.03) |
| Stroke | 3.97 (0.32-48.79) | 1.01 (0.91-1.12) | 1.56 (0.77-3.15) | 1.00 (0.98-1.02) | 1.16 (0.34-3.98) | 1.00 (0.94-1.07) |
| Digestive disease^*^ | 0.72 (0.43-1.20) | 1.00 (0.97-1.03) | 0.09 (0.01-1.65) | 1.04 (0.88-1.23) | **0.17 (0.03-0.83)**^‡^ | 1.04 (0.97-1.12) |

†: p<.01, ‡: p<.001

*Environmental disease: COPD, ASTHMA, Heart Failure, Stroke. RR: Relative Risk, Digestive disease: Control disease for study excluding peptic ulcer diseases.

a. Age standardized based on the Korea Standard Population in 2005.

b. Adjusted for seasonality, temperature, humidity, time trend, CO, SO_2_, NO_2_, PM10, and O_3_.
