# Supplementary material for: Evaluation of an air quality warning system for vulnerable and susceptible individuals in Korea: an interrupted time series analysis
Source: Epidemiol Health. 2023 Feb 14;45:e2023020. doi: 10.4178/epih.e2023020 (PMC10581892; doi:10.4178/epih.e2023020)
Supplement: Supplementary Material 3. — . Time series plot of monthly mean, age standardized emergency hospital admissions for environmental diseases by age in Korea during 2010–2019. [file epih-45-e2023020-Supplementary-3.docx]

**Supplementary Materials**

**An evaluation of the air quality warning system for vulnerable and susceptible individuals in Korea: an interrupted time series analysis**

**YouHyun Park^1,2^, Koo Jun Hyuk^2^, Hoyeon Jeong^1,2^, Ji Ye Jung^3^, Changsoo Kim^4^, Dae Ryong Kang^2,5^**

*^1^**Department of Biostatistics, Graduate School of Yonsei University, Seoul, Korea;*

*^2^National Health Big Data Clinical Research Institute, Yonsei University Wonju Industry-Academic Cooperation Foundation, Wonju, Korea*

*^3^Division of Pulmonary and Critical Care Medicine, Department of Internal Medicine, Severance Hospital, Yonsei University College of Medicine, Seoul, Korea;*

*^4^Department of Preventive Medicine, Yonsei University College of Medicine, Seoul, Korea;*

*^5^Department of Precision Medicine, Wonju College of Medicine, Yonsei University, Wonju, Korea*


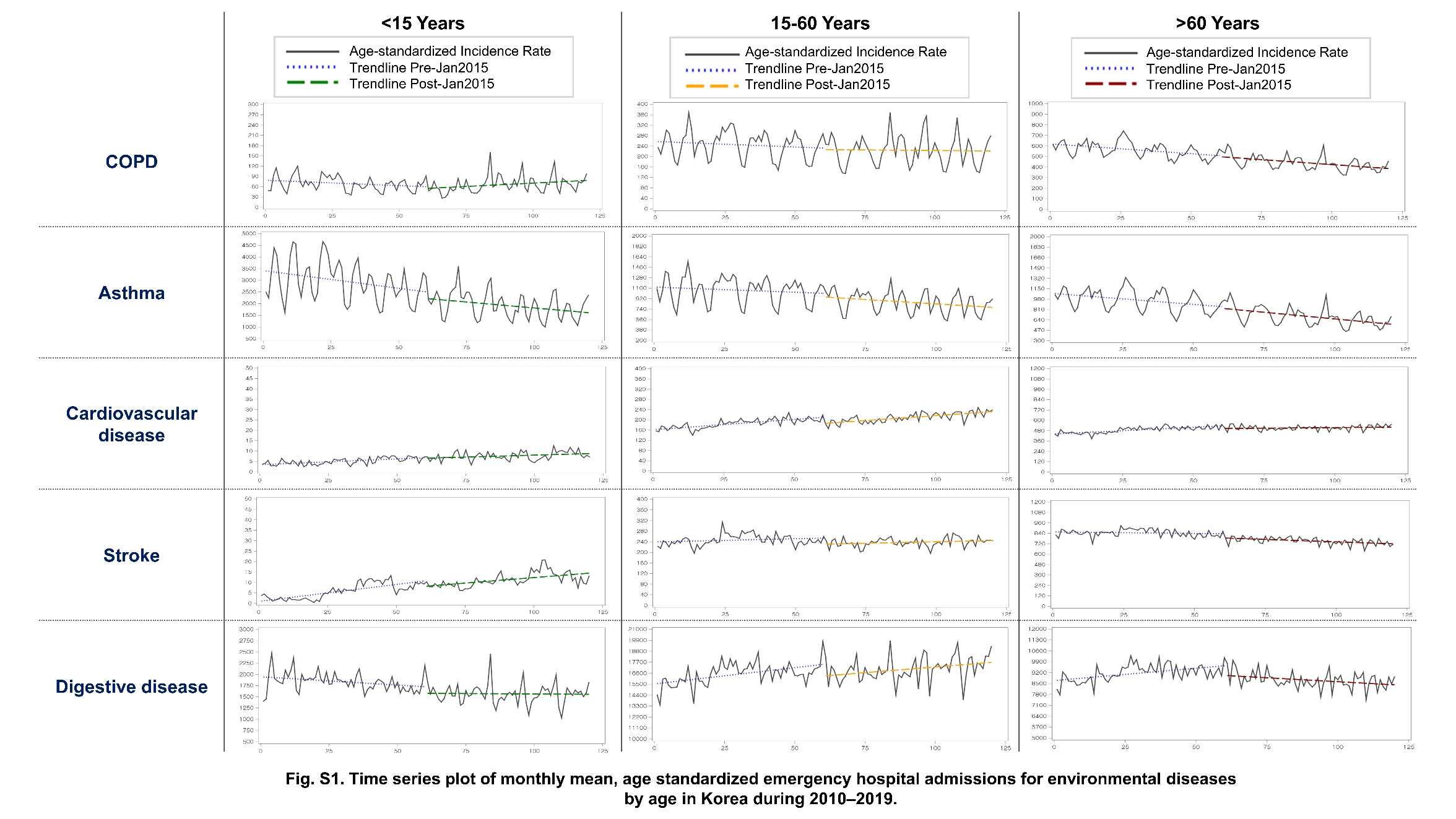


**Supplementary Material 3.** Time series plot of monthly mean, age standardized emergency hospital admissions for environmental diseases

by age in Korea during 2010–2019.
