# Supplementary material for: Evaluation of an air quality warning system for vulnerable and susceptible individuals in Korea: an interrupted time series analysis
Source: Epidemiol Health. 2023 Feb 14;45:e2023020. doi: 10.4178/epih.e2023020 (PMC10581892; doi:10.4178/epih.e2023020)
Supplement: Supplementary Material 6. — Immediate and gradual changes for incidence of environmental diseasesa after implementation of the Air Quality Warning System in multivariate analysisb by types of health care systems applicable. [file epih-45-e2023020-Supplementary-6.docx]

**Supplementary Materials**

**An evaluation of the air quality warning system for vulnerable and susceptible individuals in Korea: an interrupted time series analysis**

**YouHyun Park^1,2^, Koo Jun Hyuk^2^, Hoyeon Jeong^1,2^, Ji Ye Jung^3^, Changsoo Kim^4^, Dae Ryong Kang^2,5^**

*^1^**Department of Biostatistics, Graduate School of Yonsei University, Seoul, Korea;*

*^2^National Health Big Data Clinical Research Institute, Yonsei University Wonju Industry-Academic Cooperation Foundation, Wonju, Korea*

*^3^Division of Pulmonary and Critical Care Medicine, Department of Internal Medicine, Severance Hospital, Yonsei University College of Medicine, Seoul, Korea;*

*^4^Department of Preventive Medicine, Yonsei University College of Medicine, Seoul, Korea;*

*^5^Department of Precision Medicine, Wonju College of Medicine, Yonsei University, Wonju, Korea*

Supplementary Material 6. Immediate and gradual changes for incidence of environmental diseases^a^ after implementation of the Air Quality Warning System in multivariate analysis^b^ by types of health care systems applicable.

| Environmental diseases^*^ | **The Recipients in Medical Aid Program** | | **The Insured in National Health Insurance** | |
| --- | --- | --- | --- | --- |
|  | Immediate Effects | Gradual Effects | Immediate Effects | Gradual Effects |
|  | RR^*^ (95% CI) | RR (95% CI) | RR (95% CI) | RR (95% CI) |
| COPD | 1.03 (0.88-1.22) | 0.99 (0.98-1.00) | 1.54 (0.98-2.40) | **0.97 (0.95-0.99)** ^‡^ |
| Asthma | 1.21 (0.98-1.49) | **0.98 (0.97-0.99**) ^‡^ | **0.13 (0.04-0.50)** ^‡^ | **0.81 (0.75-0.89)** ^‡^ |
| Cardiovascular disease | **0.93 (0.88-0.99)** ^‡^ | 1.00 (0.99-1.01) | **0.66 (0.48-0.91)** ^‡^ | 1.01 (0.99-1.03) |
| Stroke | 0.97 (0.84-1.12) | 1.00 (0.99-1.01) | **0.53 (0.33-0.86)** ^‡^ | 0.99 (0.96-1.02) |
| Digestive disease^*^ | 1.16 (0.53-2.50) | 0.96 (0.91-1.00) | - | 0.99 (0.77-1.28) |

†: p<.01, ‡: p<.001

*Environmental disease: COPD, ASTHMA, Heart Failure, Stroke. RR: Relative Risk, Digestive disease: Control disease for study excluding peptic ulcer diseases.

a. Age standardized based on the Korea Standard Population in 2005.

b. Adjusted for seasonality, temperature, humidity, time trend, CO, SO_2_, NO_2_, PM10, and O_3_.
